# Association between Grip Strength and Hypertension States in Elderly Korean Individuals

**Published:** 2020-05

**Authors:** Wi-Young SO

**Affiliations:** Sports and Health Care Major, Korea National University of Transportation, Chungju-si, Korea

## Dear Editor-in-Chief

Hypertension is known to be the most prevalent and highest risk factor for cardiovascular diseases worldwide (1). Regular exercise is considered the most effective method to prevent hypertension (2). Recently, grip strength has received attention as a biomarker of health and disease status (3). Specifically, an improvement in grip strength has been found to reduce the prevalence of circulatory system diseases such as hypertension (3). Nevertheless, there are very few studies on the differences in grip strength according to hypertension status. Thus, this study aimed to examine the association between grip strength and hypertension in elderly Korean individuals.

A total of 908 elderly Korean individuals (men, 454; women, 454) aged over 65 years who participated in the 2017 Survey of National Physical Fitness, a nationally representative physical fitness test conducted by the Korea Institute of Sport Science, were analyzed in this study. In this study, the name, social security number, address, and telephone number of the participants were not included and thus the requirement for ethical approval was waived (Table 1).

**Table 1:** Participant characteristics

***Variables***	***Men (n=454)***	***Women (n=454)***
Age (yr)	74.76 ± 8.96	74.70 ± 9.06
Height (cm)	164.97 ± 6.00	152.18 ± 5.28
Weight (kg)	65.75 ± 8.58	56.52 ± 7.28
Body mass index (kg/m^2^)	24.16 ± 2.88	24.41 ± 2.95
Body fat (%)	25.85 ± 6.25	34.60 ± 6.37
Waist circumference (cm)	85.05 ± 7.51	83.04 ± 8.44
Systolic blood pressure (mmHg)	130.37 ± 13.45	128.46 ± 14.59
Diastolic blood pressure (mmHg)	74.76 ± 8.96	74.70 ± 9.06
Grip strength (kg)	33.09 ± 5.81	21.49 ± 4.27
Hypertension (number, %)	Yes 131 (28.9)	118 (26.0)
	No 323 (71.1)	336 (74.0)

Data are presented as mean ± standard deviation

Furthermore, all research procedures were controlled and approved by the Korea Institute of Sport Science.

The participants rested for more than 10 min in a sitting position. Their systolic and diastolic blood pressures in the right brachial artery were then measured by a nurse practitioner using a mercury sphygmomanometer (ALPK, Japan). According to the Joint National Committee on Prevention, Detection, Evaluation, and Treatment of High Blood Pressure-VII, hypertension is defined as a blood pressure higher than 140/90 mmHg, and a blood pressure lower than 140/90 mmHg is considered normal (4).

Grip strength was measured to assess muscular strength. The participants were asked to grip the dynamometer (TKK-5401, Japan) as tightly as possible for 2–3 seconds by placing both arms at shoulder width and gripping the dynamometer at the 2nd finger joint. The highest value recorded from two trials was 0.1 kg units. All results are presented as mean ± standard deviation. Grip strength was divided into quartiles, 1st, 2nd, 3rd, and 4th quartiles, ordered from the minimal to the maximal grip strength.

Multivariate logistic regression analyses were carried out to determine whether the grip strengths were related to hypertension, after adjustment for age and body mass index. Statistical significance was set at *P*<0.05, and all analyses were performed using SPSS ver. 18.0 (SPSS, Chicago, IL, USA).

The odd ratios (ORs) and 95% confidence intervals (CIs) for hypertension by grip strength level (with very high grip strength [4th quartile] as the reference), after adjustment for age and body mass index, were as follows: for men, 3rd quartile, 1.211 (0.622–2.359; *P*=0.573); 2nd quartile, 2.159 (1.180–3.951; *P*=0.013); and 1st quartile, 1.398 (0.756–2.586, *P*=0.285); for women, 3rd quartile, 0.799 (0.398–1.607; *P*=0.530); 2nd quartile, 0.659 (0.329–1.322; *P*=0.240); and 1st quartile, 1.889 (1.016–3.513, *P*=0.044) (Table 2).

**Table 2:** Results from multivariate logistic regression analysis for hypertension in relation to grip strength in elderly Korean individuals (n=908)

***Sex***	***Grip strength***	***Hypertension***					
		***Unadjusted OR (95% CI)***	***P-value***	***Adjusted OR (95% CI)***	**P*-value***
Men	4th quartile (Over 36.50 kg)	1.000		1.000	
	3rd quartile (33.50 kg–36.50 kg)	1.315 (0.717–2.411)	0.376	1.211 (0.622–2.359)	0.573
	2nd quartile (29.20 kg–33.40 kg)	2.162 (1.205–3.879)	0.009^**^	2.159 (1.180–3.951)	0.013^*^
	1st quartile (Under 29.20 kg)	1.374 (0.751–2.512)	0.303	1.398 (0.756–2.586)	0.285
Women	4th quartile (Over 24.30 kg)	1.000		1.000	
	3rd quartile (21.70 kg–24.30 kg)	1.447 (0.788–2.659)	0.234	0.799 (0.398–1.607)	0.530
	2nd quartile (18.90 kg–21.60 kg)	2.162 (0.440–1.628)	0.617	0.659 (0.329–1.322)	0.240
	1st quartile (Under 18.90 kg)	2.083 (1.153–3.762)	0.015^*^	1.889 (1.016–3.513)	0.044^*^

OR: odds ratio; 95% CI: 95% confidence interval

Tested using multivariable logistic regression analysis after adjustment for age and body mass index

In conclusion, among both elderly Korean men and women, the lower grip strength group showed a higher hypertension prevalence than the highest grip strength group. From this result, the grip strength in old age can be considered a predictive factor for hypertension.
